# Understanding diabetic foot

**DOI:** 10.4103/0973-3930.62596

**Published:** 2010

**Authors:** Sharad P. Pendsey

**Affiliations:** Diabetes Clinic and Research Center, ‘Shreeniwas’, Opp. Dhantoli Park, Nagpur - 440 012, India

**Keywords:** Neuropathy, peripheral vascular disease, infected foot, deformed foot

## Introduction

Diabetic foot is often quite a dreaded disability, with long stretches of hospitalization, and impossible, mounting expenses, with the ever dangling end result of an amputated limb. The phantom limb plays its own cruel joke on the already demoralized psyche. The diabetic foot, no wonder, is one of the most feared complications of diabetes.

Diabetic foot is characterized by a classical triad of neuropathy, ischemia, and infection.

Preventing the diabetic foot should be the first priority. This can be achieved by identifying the high-risk individuals, like those with peripheral neuropathy, peripheral vascular disease, foot deformities, and presence of callus.

## Epidemiology

Diabetic foot ulcers are common and estimated to affect 15% of all diabetic individuals during their lifetime. It is now appreciated that 15 – 20% of patients with such foot ulcers go on to need an amputation. Almost 85% of the amputations are preceded by diabetic foot ulcers.[1–3] Numerous risk factors for the development of foot ulcers have been suggested, the most important being peripheral sensory neuropathy followed by peripheral vascular disease. The proportion of neuropathic, neuroischemic, and purely ischemic lesions in diabetics is 54, 34, and 10%, respectively.[3] In India, it is estimated that approximately 40,000 legs are being amputated every year, of which 75% are neuropathic with secondary infection, which is potentially preventable. Certain factors, such as, barefoot walking, illiteracy, low socioeconomic status, late presentation by patients, ignorance about diabetic foot care among primary care physicians, and belief in the alternative systems of medicine contribute to this high prevalence.[4]

## Pathogenesis

Diabetic foot ulcers result from the simultaneous actions of multiple contributing causes. The major underlying causes are noted to be peripheral neuropathy and ischemia from peripheral vascular disease.

### Neuropathy

Neuropathy in diabetic patients is manifested in the motor, autonomic, and sensory components of the nervous system.[7] Damage to the innervations of the intrinsic foot muscles leads to an imbalance between flexion and extension of the affected foot. This produces anatomic foot deformities that create abnormal bony prominences and pressure points, which gradually cause skin breakdown and ulceration.

Autonomic neuropathy leads to diminished sweating. The overlying skin becomes dry and increasingly susceptible to fissures and a subsequent development of infection.

The loss of sensation as a part of peripheral neuropathy exacerbates the development of ulcerations. As trauma occurs at the affected site, patients are unable to detect the insult to their lower extremities. As a result, many wounds go unnoticed and progressively worsen as the affected area is continuously subjected to repetitive pressure and shear forces from ambulation and weight bearing. Charcot arthropathy is a consequence of peripheral neuropathy. It is the result of a combination of motor, autonomic, and sensory neuropathies, in which there is muscle and joint laxity that leads to changes in the arches of the feet. Furthermore, the autonomic denervation leads to bone demineralization via the impairment of the vascular smooth muscle, which leads to an increase in blood flow to the bone with consequential osteolysis.[8]

### Peripheral vascular disease

Although atherosclerosis in patients with diabetes is similar to that seen in nondiabetics, it is generalized, occurs prematurely and progresses at an accelerated pace. Coronary artery, cerebrovascular, and peripheral vascular disease (PVD) are the predominant manifestations of macrovascular disease in diabetes. A majority of patients with PVD have associated coronary artery disease, however, the opposite is not true.[2]

Peripheral vascular disease is found at all levels of the arterial tree but atheroma has an apparent predilection for certain sites, namely at bifurcations and bends in the artery, where hemodynamic shear stress is low or flow separation occurs. In the lower limb the common sites are the aortoiliac segment and the superficial femoral artery (SFA) in the adductor canal. In diabetics more distal vessels below the trifurcation such as the peroneal, anterior, and posterior tibials are commonly involved. Surprisingly foot vessels such as the dorsalis pedis are often spared.[2]

### Foot infection

Infection in a diabetic foot is a limb threatening condition because the consequences of deep infection in a diabetic foot are more disastrous than elsewhere mainly because of certain anatomical peculiarities. The foot has several compartments, which are inter-communicating and the infection can spread from one into another, and lack of pain allows the patient to continue ambulation further facilitating the spread. The foot also has soft tissues, which cannot resist infection, like plantar aponeurosis, tendons, muscle sheaths, and fascia. A combination of neuropathy, ischemia, and hyperglycemia worsens the situation by reducing the defense mechanism.[2]

### Osteomyelitis

Osteomyelitis generally results from a contiguous spread of deep soft tissue infection through the cortex to the bone marrow. A majority of deep, longstanding foot infections are associated with osteomyelitis. Diagnosing osteomyelitis in a patient with diabetic foot is often difficult. Major problems include differentiating soft tissue infection from bone infection and infections from non-infectious disorders (Charcot Foot). Plain radiography usually shows focal osteopenia, cortical erosions or periosteal reaction in the early stage and sequestration in the late stage. A simple clinical test is probing to the bone. A sterile metal probe is inserted into the ulcer if it penetrates to the bone it almost confirms the diagnosis of osteomyelitis. Chronic discharging sinus and sausage-like appearance of the toe are the clinical markers of osteomyelitis. Definitive diagnosis requires obtaining a bone biopsy for microbial culture and histopathology.

The newer imaging techniques are nuclear bone scan, computerized tomography scan (CT), positron emission tomography (PET), and magnetic resonance imaging (MRI). Of these, MRI is more sensitive and specific.[9]

## Classification

Diabetic foot is classified into two major types.

1. The Neuropathic Foot where neuropathy dominates
2. The Neuroischemic Foot, where occlusive vascular disease is the main factor, although neuropathy is present.

Neuropathy leads to fissures, bullae, neuropathic (Charcot) joint, neuropathic edema, and digital necrosis. Ischemia leads to pain at rest, ulceration on foot margins, digital necrosis, and gangrene. Differentiating between these entities is essential because their complications are different and they require different therapeutic strategies.[10]

The University of Texas Wound Classification System is the most acceptable classification. [Table 1].[11]

**Table 1:** Wound classification system[11]

Stages	Description
Stage A	No infection or ischemia
Stage B	Infection present
Stage C	Ischemia present
Stage D	Infection and ischemia present
Grading	
Grade 0	Epithelialized wound
Grade 1	Superficial wound
Grade 2	Wound penetrates to tendon or capsule
Grade 3	Wound penetrates to bone or joint

The older classification, suggested by Wagner,[12] accounts only for wound depth and appearance and does not consider the presence of ischemia or infection.

## Examination of feet

Examination of the feet is an integral part of the physical examination of every patient, more so a diabetic patient. One should look for neuropathic changes like dry skin, fissures, deformities, callus, abnormal shape of foot, ulceration, prominent veins, and nail lesions. Careful attention should be given to the interdigital spaces. Significant ischemia is characterized by loss of hair on the dorsum of the foot and a dependent rubor. One should feel the foot for warmth or coldness; examine the peripheral pulsations such as dorsalis pedis, which can be felt lateral to the exensor hallucis longus tendon and posterior tibial, which is above and behind the medial malleolus. The femoral artery should also be palpated and auscultated for the presence of bruit. The plantar aspects of the feet should be felt for the presence of any bony prominence or callus.

Sensory neuropathy can be tested by using monofilaments and biothesiometry. If these are not available, the detection of light touch by cotton wool, pinprick, and vibration sense using a 128 Hz tuning fork is sufficient. The goal is to detect whether the patient has lost protective sensations (LOPS), rendering him susceptible to foot ulceration.

A hand-held Doppler can be used to confirm the presence of pulses and to quantify the vascular supply. When used together with a sphygmomanometer, the ankle and brachial systolic pressures can be measured and the ratio then calculated. In normal subjects, the ankle systolic pressure is higher than the brachial systolic pressure. The normal ABI > 1, in the presence of ischemia it is < 0.9. Absent or feeble pulses, with ABI < 0.9, confirm ischemia. Conversely, the presence of pulses and ABI > 1 rules out significant ischemia.[2]

## Management

Diabetic foot should be managed using a multidisciplinary team approach.

The management of diabetic foot ulcers includes several facets of care. Offloading and debridement are considered vital to the healing process, for diabetic foot wounds.[13] The goal of offloading is to redistribute force from the ulcers sites and pressure points at risk, to a wider area of contact. There are multiple methods of pressure relief, including total contact casting, half shoes, removable cast walkers, wheelchairs, and crutches.[14]

An open diabetic foot ulcer may require debridement if necrotic or unhealthy tissue is present. The debridement of the wound will include the removal of the surrounding callus, which decreases the pressure points at the callused sites on the foot. Additionally, the removal of unhealthy tissue can aid in removing colonizing bacteria in the wound. It will also facilitate the collection of appropriate specimens for culture and permit examination for the involvement of deep tissues in the ulceration.[15]

Infection in a diabetic foot is limb threatening and at times life threatening, and therefore, must be treated aggressively. Superficial infections should be treated with debridement, oral antibiotics, and regular dressings. Deep infections are considered when the signs of infection are combined with evidence of involvement of deeper tissue structures such as bones, tendons or muscles. Although superficial infections are usually caused by gram-positive bacteria, the deep foot infections are invariably polymicrobial and caused by gram-positive bacteria, gram-negative bacteria, and anaerobes. All patients with deep infections should be hospitalized and started on broad-spectrum antibiotics.[16] The choice of antibiotics initially should be empirical, but once the culture reports are known, it should be specific and narrowed down. Surgical debridement should be carried out, which should include all the devitalized tissues, sloughed tendons, and infected bones.

Multiple injections of insulin or continuous insulin infusion should be instituted to achieve metabolic control.

### Dressing material

The selection of wound dressings is also an important component of diabetic wound care management. Saline-soaked gauze dressings, for example, are inexpensive, well-tolerated, and contribute to an atraumatic, moist wound environment.

A wide variety of new dressing materials have been developed.

Some of the newer dressings are – film dressing, foam dressing, non-adherent dressings, hydrogels, hydrocolloids, and alginates.

The treating foot care team has to make an appropriate choice of dressing for a particular type of wound.[17,18]

A number of adjunctive wound care treatments are under investigation and in practice for treating diabetic foot ulcers. The use of human skin equivalents has been shown to promote wound healing in diabetic ulcers via the action of cytokines and dermal matrix components that stimulate tissue growth and wound closure.[19,20] A recombinant platelet-derived growth factor is also currently in use and has been shown to stimulate wound healing. It is a recombinant human PDGF-BB gel preparation, which is used for non-infected neuropathic ulcers. It is spread over the wound and covered with non-adherent, saline-soaked gauze dressing. The dressing is changed once or twice every day. It has to be realized that this gel therapy is effective only if other modalities such as recurrent surgical debridement of the ulcer and offloading are adhered to.[21]

### Revascularization

Patients with evident peripheral ischemia need revascularization as adequate arterial blood supply is necessary to facilitate wound healing and resolve the underlying infection.

Surgical bypass is a common method of treatment for ischemic limbs, and favorable long-term results have been reported. Up to a 90% 10-year limb salvage rate has been demonstrated with surgical bypass procedures of the lower extremity.[22,23] In cases where there are multiple levels of occlusion, revascularization at each point is necessary to restore the arterial blood flow and increase the chance for limb salvage. Transluminal angioplasty of the iliac arteries in conjunction with a surgical bypass in the distal extremity may be implemented, and efficacy has been demonstrated in diabetic patients.[22]

Transluminal angioplasty is also an excellent option for single stenotic lesions.

For multiple lesions or occlusions > 15 cm or occlusion of infra-popliteal vessels, bypass surgery is the best option.[24]

## Prevention

Early detection of potential risk factors for ulceration can decrease the frequency of wound development. It is recommended that all patients with diabetes undergo a foot examination at least annually, to determine the predisposing conditions to ulceration.

Patients should be educated regarding the importance of maintaining good glycemic control, wearing appropriate footwear, avoiding trauma, and performing frequent self-examinations.

### Prevention of diabetic foot includes:

1. Primary prevention: Screening of high risk feet and proper advice on preventive footwear
2. Secondary prevention: Management of trivial foot lesions such as callus removal, treatment of nail pathologies, deroofing blisters, and so on.
3. Tertiary prevention: Prompt referral to a specialist for advanced foot lesions.[4]

Significant limb salvage and prevention of amputation is achieved by training of primary care physicians and their paramedics in diabetic foot care. The Step by Step Project of improving diabetic foot care was recently executed by the author under the auspices of the World Diabetes Foundation.[4]

Patient education and lifelong surveillance are essential to protect feet at risk from ulceration. Patients need to realize that high risk feet need to be used sparingly. The activity level should be as minimal as possible.

Understanding the diabetic foot, proper examination of feet, investigations to classify the foot ulcers, and proper management techniques using a team approach, along with preventive steps, would go a long way in limb salvage and prevention of limb amputation in people with diabetes.
